# Seeking But Not Discussing Online Health Information With Physicians: Cross-Sectional Survey Study of eHealth Literacy–Empowerment Profiles and Patient-Centered Communication

**DOI:** 10.2196/78836

**Published:** 2026-02-18

**Authors:** Qianfeng Lu, Wen Jiao, Angela Chang, Peter Johannes Schulz

**Affiliations:** 1 Faculty of Communication, Culture and Society Università della Svizzera italiana Lugano Switzerland; 2 School of Communication Soochow University Soochow China; 3 Faculty of Social Sciences University of Macau Macau China; 4 Department of Communication and Media Ewha Womans University Seoul Republic of Korea

**Keywords:** empowerment, literacy, patient-provider communication, patient-centered communication, health information, misinformation

## Abstract

**Background:**

Patients frequently search for health information online and value physician support in evaluating and interpreting their findings, yet many hesitate to share their online searches with their physicians. This hesitation hinders shared decision-making and compromises patient care. While extensive research has examined patients’ online health information–seeking behaviors, little has focused on patients’ disclosure of this information to their physicians during consultations.

**Objective:**

Guided by the Health Empowerment Model and the Linguistic Model of Patient Participation in Care, this study aims to (1) identify distinct patient profiles based on eHealth literacy and psychological health empowerment levels, (2) examine how these patient profiles differ in online health information seeking and disclosure to physicians, and (3) investigate whether patient-centered communication (PCC) promotes information disclosure and whether this effect varies by patient profile.

**Methods:**

This cross-sectional study surveyed 2001 Chinese participants recruited through convenience sampling. Patient profiles were identified using *k*-means cluster analysis with standardized *z* scores of eHealth literacy and psychological health empowerment. Differences between profiles in information behaviors were examined using 1-way Welch ANOVA, chi-square tests, and pairwise comparisons. Regression analyses examined the association between PCC and disclosure of online health information. Moderation analyses using the Hayes PROCESS macro assessed whether this association varied across patient profiles.

**Results:**

Four distinct patient profiles were identified: effective self-managers (996/2001, 49.8%), moderate-needs dependent patients (408/2001, 20.4%), high-needs patients (68/2001, 3.4%), and dangerous self-managers (529/2001, 26.4%). Profiles differed significantly in information-seeking intentions (*F*_3,289_=62.09; *P*<.001; η²=0.12) and disclosure intentions (*F*_3,299.41_=66.08; *P*<.001; η²=0.09). “Effective self-managers” showed the highest seeking (mean 4.01, 95% CI 3.96-4.06) and disclosure intentions (mean 3.43, 95% CI 3.36-3.50), while “high-needs patients” showed the lowest intentions for both behaviors. Actual information-seeking rates also differed significantly across profiles (*χ*²_3_=103.4; *P*<.001), with “effective self-managers” having the highest rate (800/996, 80.3%) and “high-needs patients” the lowest (25/68, 36.8%). Among seekers, disclosure rates varied significantly (*χ*²_3_=23.1; *P*<.001), with “high-needs patients” showing the highest disclosure (16/25, 64%) despite having the lowest seeking rate. PCC was positively associated with actual information disclosure behavior (odds ratio 1.26, 95% CI 1.04-1.53; *P*=.02), with no significant moderation by patient profiles (*χ*²_3_=1.7; *P*=.64).

**Conclusions:**

This study extends existing literature from information-seeking behavior to patients’ disclosure of online findings to physicians. Unlike prior research that examined eHealth literacy and psychological health empowerment separately, this study integrated these constructs to identify meaningful patient profiles with distinct information behavior patterns. PCC facilitates disclosure regardless of patient profile. For practice, physicians should adopt a PCC that acknowledges patients’ online research efforts, promoting safer information use and stronger patient-physician relationships.

## Introduction

### Background

The use of digital platforms and online information channels is becoming increasingly common for patients seeking health-related information [1,2]. Accessing online health information offers various benefits to patients. By seeking health information online, patients can gain social support, be better informed, and become more prepared to participate in their health care [3-5]. However, drawbacks exist. Online searches may increase patient anxiety, create confusion, and even lead to medical nonadherence [6,7]. The risks are associated with the potential misuse of online health information [8,9], as a significant portion of online content contains misinformation [10] or may be incomplete or unsuitable for a patient’s specific condition [11].

Physicians can play a crucial role in guiding patients to navigate online health information [12]. Patient perspectives showed that they generally want physicians to help them understand and assess the suitability of online health information for their specific condition [4,13]. Medical consultations provide valuable opportunities for patients to receive professional advice tailored to their specific conditions. When physicians discuss online health information with patients during medical encounters, they can identify and possibly correct misconceptions patients acquired through internet searches, potentially preventing the misuse of online materials [14]. Additionally, when physicians and patients discuss internet-sourced health information, it helps doctors better understand patients’ perceptions of their conditions and health care needs [15]. This understanding, even when it reveals discrepancies between professional medical advice and patients’ personal beliefs, creates opportunities for forming more engaged and shared decision-making [14]. Interview studies have shown that conversations about internet-sourced information can enhance patient engagement during consultations [16], strengthen the physician-patient relationship [17], and foster mutual trust [18].

Despite these benefits, many patients still withhold internet-sourced health information from their physicians [19,20]. Barriers to discussing online findings include limited consultation time, concerns about online information quality (such as conflicting or inaccurate content), and patients’ perceptions that physicians may be unwilling to engage in such discussions [21,22]. With patients increasingly turning to online resources as their first source of health information prior to consultations [23], it is essential to understand not only their search behaviors but also what influences their decision to disclose or withhold these findings during medical encounters. However, the existing literature has primarily focused on search behaviors while largely neglecting the disclosure aspects [21].

This study examines patients’ online health information seeking and their disclosure of these findings to physicians during medical encounters. We draw on the Health Empowerment Model (HEM) [24] to understand how patient characteristics shape information behaviors, and the Linguistic Model of Patient Participation in Care [25] to examine how physician communication influences disclosure.

### Theoretical Framework

#### Health Empowerment Model

The HEM recognizes the distinct but complementary roles of psychological health empowerment and health literacy in influencing health behaviors [24]. Health literacy refers to the ability to access, understand, and evaluate health information [26], and psychological health empowerment is more about the patient’s sense of control, confidence, and motivation to act actively in managing their health [27]. Without literacy skills, patients may struggle to identify reliable health information; without empowerment, even accurate information may go underused [24]. Together, these two factors enable patients to make informed decisions and actively manage their health [24]. This model is especially valuable in the digital age, where the use of online health information has increased rapidly, intensifying the need for patients to possess both the literacy skills to accurately evaluate online information (eHealth literacy) and the motivation and confidence to seek and use it (psychological health empowerment) [28]. Depending on patients’ ability to navigate online content, online health information can empower but may also misinform them [29].

The HEM delineates 4 distinct combinations of empowerment and health literacy, each of which can be associated with health information behaviors uniquely [30]. The “effective self-manager” represents the ideal scenario: an empowered patient with high eHealth literacy who proactively seeks out and acts on that online health information, making informed decisions based on their ability to evaluate information accurately. In contrast, the “dangerous self-manager” is empowered but has low eHealth literacy. They are proactive in seeking out online information, yet lack the skills to evaluate it accurately, which may lead to poor or even harmful health decisions [24]. The “needlessly dependent patient” has the high literacy skills to understand online health information but lacks the empowerment to act on it, often deferring decisions to others despite having the knowledge to engage more actively in their care. Finally, the “high-needs patient” is deficient in both empowerment and eHealth literacy, typically taking a passive role in their health care and requiring support to find, understand, and apply online health information [24].

The HEM has been applied to examine patient health status [31], help-seeking behaviors [32], medical decision-making [33], chronic disease management [34,35], and participation in online health communities [36]. These studies consistently demonstrate that patients require both high empowerment and health literacy to effectively manage their health and use health resources, including online health information [37]. Deficiencies in either factor lead to suboptimal health behaviors and poorer outcomes. The interplay between empowerment and literacy creates different patient profiles with varied ways of managing health, seeking information, and engaging with health care providers [24]. Building on this framework, this study aims to identify distinct patient categories based on the combination of these two factors in the context of online health information behaviors.

#### eHealth Literacy

The traditional concept of health literacy is defined as the personal cognitive and social skills to access, understand, and use health information. eHealth literacy extends it by emphasizing the knowledge and competencies required to navigate electronic information platforms [38-40]. The increasing prevalence of health information and proliferation of misinformation on the internet have made these skills more urgent than ever [41]. Individuals with higher eHealth literacy are more likely to seek health information online [42,43]. For example, empirical studies have found that individuals with higher levels of eHealth literacy are more likely to use health applications to acquire information [44,45], access social media platforms to find and share health-related content [43], and explore national health information portals for health care [46].

Patients’ eHealth literacy skills shape both the quality of information they find online and their communication with health care providers [47,48]. A systematic review found that patients with low health literacy had more difficulty evaluating online health information and showed less trust in such information [29]. Conversely, those with higher literacy communicate more clearly with providers as they can articulate their problems and information needs more precisely [47]. While these review findings were based on general health literacy, they likely extend to eHealth literacy, given that fundamental health literacy skills are also core components of eHealth literacy [39]. Empirical findings on whether eHealth literacy leads patients to disclose their internet findings with providers are mixed: some studies show a positive association [40], whereas others report no significant link [49]. These inconsistencies indicate that, beyond the ability to handle online information, a motivational driver may also be important to consider. Drawing on the HEM, we include psychological health empowerment as a complementary determinant to examine alongside eHealth literacy.

#### Psychological Health Empowerment

A concept that often intertwines with health literacy is psychological health empowerment [50]. Psychological health empowerment is also frequently associated with online health information behavior but often studied separately from eHealth literacy [4]. It is sometimes referred to as health empowerment or patient empowerment [27,51]. Health empowerment has been consistently associated with more active online health information seeking. Patients view online information-seeking as a means to be informed and prepared to manage their health conditions [4]. By accessing online health resources, patients perceive greater control over their conditions [52] and increased confidence when communicating with physicians during medical consultations [53,54]. In essence, while empowered patients are more likely to seek information, the act of seeking and finding relevant health information online further enhances their sense of empowerment [37].

Health empowerment also determines how information is integrated into health decision-making [55]. When patients feel empowered, they actively engage in decision-making with health care providers [56]. This sense of empowerment motivates patients to take an active role in managing their health, including seeking and using online health resources. Empowered patients are more likely to bring their online findings into clinical discussions with physicians as a means to address their information needs, clarify uncertainties, and participate in decision-making [57]. In contrast, patients with low empowerment often remain passive in health care interactions with their providers [57,58]. Despite potentially having found the same information online, these less-empowered patients may hesitate to disclose their findings during consultations, instead deferring entirely to physician direction [24].

To sum up, eHealth literacy equips patients with the skills needed to critically assess the reliability and relevance of online content they encounter [39]. Psychological health empowerment, on the other hand, reflects patients’ motivation and confidence to act on this information and actively participate in their health care [59]. The HEM posits that both eHealth literacy and psychological health empowerment are necessary for effective use of health information and that their combination determines how patients engage with health information. Although previous research has examined these two concepts, they were typically studied separately, with studies often focusing on either health literacy or health empowerment but failing to integrate both [37,60]. To fill this gap, this study also examines health information behaviors across different eHealth literacy and empowerment profiles, from online searching before consultations to information sharing during physician encounters.

#### Patient-Centered Communication

Despite recognizing the potential benefits of discussing online health information with physicians, many patients hesitate to share their internet findings during consultations [21]. This reluctance stems largely from patients’ concerns about how physicians will react to their online search [22,61]. Empirical evidence demonstrates that patients’ hesitation to disclose internet-sourced information is directly related to their communication experience with physicians [4].

Patient-centered communication (PCC)—defined as a clinical communication approach where providers seek to understand patients’ perspectives, reach shared understanding, and involve patients as partners in care decisions [62]—offers a promising approach to encourage information sharing. It differs fundamentally from the paternalistic model by positioning patients as equal participants in the clinical relationship. Research shows that PCC promotes patient health care participation and is linked to improved patient trust and satisfaction with providers [63,64]. When physicians adopt patient-centered approaches, patients feel their perspectives are valued [65] and are more likely to share internet-sourced information [21]. Conversely, when patients perceive physicians as unreceptive or threatened by their online research, they are unwilling to disclose [21,52,66].

The importance of physician communication is supported by the Linguistic Model of Patient Participation in Care [25]. This model identifies 3 key determinants of patient participation in medical encounters: predisposing factors, enabling factors, and physician communication behaviors. Predisposing factors encompass patients’ sociodemographic and psychosocial characteristics, including psychological health empowerment, that influence their propensity to participate actively in health care encounters. Enabling factors refer to patients’ communicative resources and capabilities, such as eHealth literacy skills, that determine their capacity to engage effectively with health care providers and articulate their needs. Physician communication behaviors include verbal and nonverbal practices, such as providing clear explanations and demonstrating empathy, that can either facilitate or inhibit patient participation. The model posits that these 3 factors interact rather than operate independently [25]. It suggests that even patients with high empowerment and literacy may withhold information if physician communication does not support their disclosure. Thus, PCC serves as a crucial contextual factor that can affect information disclosure, regardless of patients’ individual characteristics.

PCC may not affect all patients equally. The HEM suggests that patients with different literacy and empowerment levels rely on their physicians differently [24]. Those with high empowerment and literacy may already feel confident sharing online findings, while patients with lower empowerment and literacy may be more sensitive to physician communication styles and depend more heavily on physician encouragement. This raises the question of whether the effect of PCC on disclosure varies across patient profiles. Therefore, the final aim of this study is to examine the impact of PCC on patient information disclosure and whether it varies across different patient profiles.

Based on the above, this study poses the following research questions (RQs):

- RQ1: What distinct patient profiles can be identified based on eHealth literacy and psychological health empowerment?
- RQ2: How do patients’ online information-seeking behaviors prior to consultations and their subsequent disclosures to physicians differ across the identified patient profiles?
- RQ3a: How does patient-centered communication relate to patients’ disclosure of online health information to physicians? RQ3b: How does this relationship differ across patient profiles?

## Methods

This cross-sectional survey study is reported according to CHERRIES (Checklist for Reporting Results of Internet e-Surveys) guidelines [67].

### Recruitment

This study focuses on China, the world’s largest digital community, with approximately 1.08 billion internet users in 2023 [68]. This cross-sectional survey study was conducted as part of a larger research project examining health information-seeking behavior and health care use in China.

Data collection was conducted in Mainland China and Macau (Special Administrative Region of China) between September and November 2022. The survey was distributed online through Chinese social media platforms, including WeChat, QQ, and Sina Weibo. The survey was created using the Qualtrics platform and took approximately 15 minutes to complete.

Eligible participants were required to be Chinese, aged 18 years or older, and residing in Mainland China or Macau. Snowball convenience sampling was used. Initial recruitment began on the University of Macau campus, where research team members approached individuals in public areas (eg, libraries and classrooms) and invited them to participate. Participants who completed the survey were asked to share it with their friends and family members who met the eligibility criteria.

A total of 3387 respondents answered the survey. The final analytical sample included 2001 participants who provided complete responses on all focal variables (eHealth literacy, health empowerment, and information seeking and disclosure behavior). Among these, 1569 participants had received health care in the previous year and could provide responses regarding physician communication, forming the subsample for PCC analyses. No formal sample size calculation was conducted, and the sample size was determined by the number of complete responses obtained during the data collection period. Survey design and recruitment procedures are also published in Jiao et al [37].

### Ethical Considerations

This study received ethical approval from the University of Macau’s Ethical Committee for the Social Sciences and Humanities (application code: SSHRE22-APP093-FSS), which covered the complete data collection procedure. The original ethical approval included permission for secondary analyses of the collected data; therefore, no additional approval was required for this study. All participants provided informed consent prior to completing the survey. Participants were informed that the study aimed to help researchers and policymakers better understand population health behaviors related to internet use for health purposes. Participant privacy and confidentiality were protected through anonymous data collection. No personally identifiable information was collected, and all survey responses were deidentified before data analysis. No compensation was provided to participants. Participation was entirely voluntary, and participants could withdraw at any time without consequence. No images or materials in this manuscript contain identifiable participant information.

### Measures

#### eHealth Literacy

The eHealth Literacy Scale (eHEALS), a validated 8-item instrument developed by Norman and Skinner [69], was used to evaluate participants’ eHealth literacy. It uses the operational definition of eHealth literacy as “an individual’s ability to seek, find, understand, appraise, and apply health information from electronic sources to address or solve a health problem” and uses self-reported measures to assess perceived competence across these domains. Despite being developed nearly two decades ago, eHEALS continues to receive research attention with recent validation studies confirming its robustness across diverse populations [70,71]. The core competencies it measures (seeking, understanding, appraising, and applying health information from electronic sources) remain essential regardless of the online information source and represent foundational skills needed to navigate today’s digital health information landscape.

We used the Chinese translation of eHEALS validated by Chang and Schulz [72], which demonstrated good psychometric properties in Chinese populations and has been empirically applied in studies among Chinese-speaking populations [73,74]. Responses were measured on a 5-point Likert scale, with higher scores indicating greater eHealth literacy. The mean of all item scores was calculated to represent participants’ eHealth literacy levels. The reliability of eHEALS was assessed using Cronbach α. Additionally, we reported McDonald ω, which is less affected by the number of items (a limitation of Cronbach α) and does not assume equal factor loadings of items. Both reliability tests showed good internal consistency (Cronbach α=0.93; McDonald ω=0.93). The complete survey wording can be found in Multimedia Appendix 1.

#### Psychological Health Empowerment

The Psychological Health Empowerment Scale (PHES) was used to measure patient empowerment [27]. This established 8-item instrument assesses individuals’ perceived control over one’s health, perceived competence of understanding of one’s health condition, and their motivation to achieve health goals. Sample items include statements such as “I can motivate myself to manage my health and make a better life” and “I can make every possible effort to achieve health goals.” The PHES has been widely adopted across diverse populations to measure health empowerment [75]. We used the Chinese translation by Jiang and Street [76], which has subsequently been used in other studies with Chinese-speaking populations [77,78]. Responses were recorded on a 5-point Likert scale, with higher scores indicating a greater sense of health empowerment. The overall health empowerment level was calculated by averaging the 8 items (Cronbach α=0.90; McDonald ω=0.90).

#### Patient-Centered Communication

Participants who had received health care in the preceding year (n=1569) were asked about their communication experiences with physicians. The 7-Item Patient-Centered Communication Scale based on Epstein and Street’s [79] model was used to assess how frequently physicians (1) provided opportunities to ask health-related questions, (2) paid attention to feelings and emotions, (3) involved patients in health care decisions to their desired extent, (4) ensured understanding of health management tasks, (5) explained medical information comprehensibly, (6) spent adequate time with patients, and (7) helped manage health-related uncertainty. This scale has been widely used to estimate PCC [80,81]. We used the Chinese translation from the Health Information National Trends Survey in China (HINTS-China) [82]. Responses were recorded on a 5-point Likert scale. The overall patient-physician communication was calculated by averaging the 7 items, with higher scores indicating better PCC (Cronbach α=0.89; McDonald ω=0.89).

#### Information Seeking and Disclosure Behavior

The two facets of participants’ health information-seeking behavior in the clinic context, intended versus actual, were assessed. We inquired about their past behaviors regarding whether participants have sought out health information on the internet before consulting with a doctor (actual seeking) and whether such information was subsequently discussed during medical visits (actual disclosure). The two specific survey questions are “Reflecting on your recent medical consultations, did you search for health information on the Internet about your condition before meeting with your doctor?” For those who answered positively (n=1428), they were further queried, “Furthermore, did you discuss the information you found on the Internet with your doctor during the consultation?” A binary response of “yes” (=1) or “no” or “I am not sure” (=0) was applied.

The hypothetical scenarios aimed to elicit intended behaviors. Participants were presented with a situation in which they hypothetically experienced a new health symptom (eg, chest pain) and were asked to predict their likelihood of searching for health information online before a scheduled doctor’s appointment (intended seeking) and their propensity to share the found information with the doctor (intended disclosure). The corresponding survey questions were as follows: “Imagine experiencing chest pain for one week, a symptom you’ve never had before. As a result, you decide to schedule a medical appointment with a new doctor. How likely are you to search for information about your condition on the Internet before visiting the doctor? And how likely are you to share the information you found on the Internet with the doctor?” A 5-point Likert scale ranging from “very unlikely” (=1) to “very likely” (=5) was used. A pilot study with university adults in Macau and feedback from the pilot study were used to refine these survey questions.

#### Control Variables

We collected participants’ demographic information, including age, gender, and education level. Age was recorded in years, while education level was categorized based on the highest grade completed, ranging from primary school and below (coded as 1) to a bachelor’s degree and above (coded as 6). Gender was represented as a binary variable, with “female” coded as 0. In addition, self-rated general health status and trust in internet-sourced health information were included as control variables to minimize confounding effects. Participants were asked to rate their overall health status on a scale from 1 (poor) to 5 (excellent). Trust in health information from social media, websites, search engines, and apps was measured with a 5-point scale (“1=not at all” to “5=a lot”). The mean scores of these trust responses were calculated to provide an overall trust in online health information (Cronbach α=0.89; McDonald ω=0.80).

### Statistical Analysis

Descriptive statistics were conducted to ascertain the overall sample characteristics. Then, a *k*-means cluster analysis was performed using the standardized *z* scores of 2 key variables: eHealth Literacy and Psychological Health Empowerment. This analysis was carried out in the R program (R Foundation for Statistical Computing), using the “NbClust” package to identify the optimal number of clusters [83]. The NbClust package determines the optimal number of clusters by computing different cluster validity indices, such as the Silhouette index, Dunn index, and Calinski-Harabasz criterion. Rather than relying on a single criterion, it evaluates multiple indices and provides a consensus solution based on the majority rule. NbClust has been widely used in previous empirical studies across various disciplines. Following the cluster analysis, the distinct patient eHealth Literacy and Empowerment Profiles (eHL-E Profiles) were established.

Then, 1-way ANOVA, using Welch correction to account for potential heterogeneity of variances, was conducted to examine differences in intended information-seeking and disclosure behaviors across the different eHL-E Profiles. Games-Howell post hoc comparisons were performed to identify specific between-group differences. Additionally, chi-square tests were used to assess differences in actual information-seeking and disclosure behaviors among the eHL-E Profiles. Pairwise comparisons with Bonferroni adjustment were further conducted to identify which specific eHL-E Profiles differed significantly from each other in their actual information behaviors.

Finally, regression analyses were performed to examine the association between PCC and patient information disclosure. For the dichotomous outcome actual disclosure (0=no, 1=yes), we fitted a binary logistic regression with PCC as the focal predictor. For the continuous outcome intended disclosure (interval scaled), we ran a multiple linear regression. All models controlled for eHealth literacy, health empowerment, self-rated health, overall trust in online health information, and demographic variables (age, gender, and education) to mitigate confounding bias. To test whether the effect of PCC on disclosure varied by patient profile groups, we estimated a simple moderation model (model 1) in the Hayes PROCESS macro (version 4.3), controlling for self-rated health, trust in online health information, and demographic variables. We requested 5000 bootstrap samples to obtain bias-corrected 95% CIs for all conditional effects.

## Results

### Sample Characteristics

Descriptive statistics are displayed in Table 1. The mean age of participants was 31.78 (SD 13.58) years, 64.3% (1202/1870) were female, and 82.5% (1508/1829) had received a college degree or higher education. General health status was rated, on average, as good (mean 2.98, SD 0.81). Overall trust in online health information was moderate (mean 2.48, SD 0.69).

**Table 1:** Demographic and health-related characteristics of study participants (N=2001).

Variables			Values
Age^a^ (years), mean (SD)			31.78 (13.58)
**Gender^b^** **, n (%)**			
	Female	1202 (64.3)	
	Male	668 (35.7)	
**Education^c^**			
	Mean (SD)	4.60 (1.07)	
	Primary school or below, n (%)	24 (1.3)	
	Junior middle school, n (%)	58 (3.1)	
	High school, n (%)	239 (13.1)	
	Junior college, n (%)	258 (14.1)	
	Bachelor’s degree, n (%)	982 (53.7)	
	Master’s degree or above, n (%)	268 (14.7)	
Health status, mean (SD)			2.98 (0.81)
Trust in online health information, mean (SD)			2.48 (0.69)
eHealth literacy, mean (SD)			3.48 (0.70)
Health empowerment, mean (SD)			3.77 (0.59)
Patient-centered communication^d^, mean (SD)			2.53 (0.63)
Intended seeking, mean (SD)			3.79 (1.01)
Intended disclosure, mean (SD)			3.19 (1.09)
Actual seeking (yes), n (%)			1428 (71.4)
Actual disclosure^e^ (yes), n (%)			800 (56.0)

^a^Among the total sample of 2001 participants, missing data were observed for 139 participants.

^b^Among the total sample of 2001 participants, missing data were observed for 131 participants.

^c^Among the total sample of 2001 participants, missing data were observed for 172 participants.

^d^A total of 1569 participants who had received health care in the previous year provided answers regarding their communication with the physician.

^e^A total of 1428 participants who had searched for health information on the internet before meeting their physicians responded about whether they had discussed this information with their physician.

### Patient Profiles Based on eHealth Literacy and Psychological Health Empowerment

RQ1 asked what distinct profiles of patients could be identified based on their eHealth literacy and psychological health empowerment. *K*-means cluster analysis identified a 5-cluster solution as the optimal fit. Two clusters exhibited high scores in both eHealth literacy and health empowerment. Specifically, one had mean scores for eHealth literacy and health empowerment of 4.27 (95% CI 4.21-4.34, SD 0.51) and 4.61 (95% CI 4.57-4.65, SD 0.31), respectively; another had corresponding scores of 3.90 (95% CI 3.88-3.92, SD 0.28) and 3.89 (95% CI 3.87-3.90, SD 0.23). Aligned with the HEM, these clusters exemplify a group with both high literacy and high empowerment, and can be labeled as the “effective self-manager.” Therefore, they were amalgamated into a single profile, profile 1, which encompasses 996 participants (996/2001, 49.8%). This aggregated profile demonstrated mean eHealth literacy and health empowerment scores of 3.98 (95% CI 3.96-4.01, SD 0.38) and 4.05 (95% CI 4.02-4.07, SD 0.39), respectively, signifying high levels of both eHealth literacy and empowerment.

**Figure 1:**
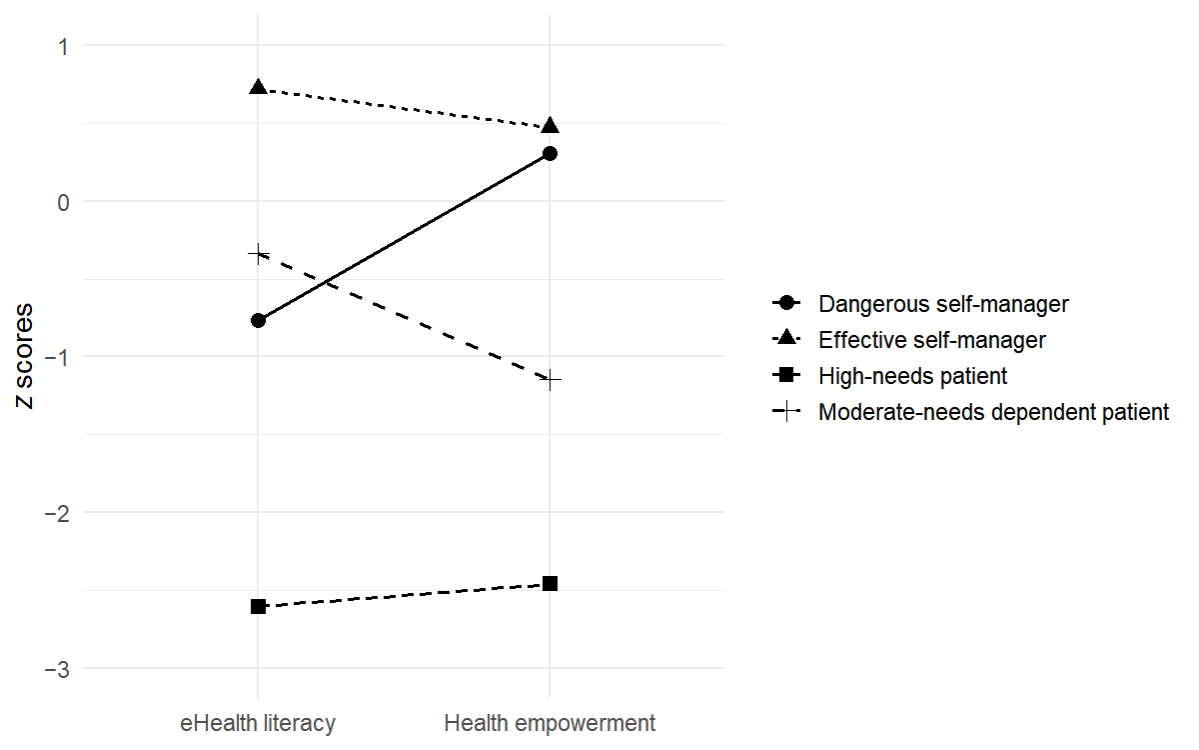
Standardized z scores for eHealth literacy and psychological health empowerment by patient profile.

The remaining 3 clusters exhibited distinct combinations of eHealth literacy and empowerment levels, and thus we retained these unique eHealth literacy–empowerment profiles without merging. Figure 1 displays the *z* scores for eHealth literacy and health empowerment across the 4 profiles ultimately identified, including the aggregated profile 1, the effective self-manager. Table 2 lists their mean scores.

Profile 2 (408/2001, 20.4%) exhibited a comparatively low level of eHealth literacy (mean 3.25, 95% CI 3.21-3.29, SD 0.41) and an even lower level of empowerment (mean 3.09, 95% CI 3.06-3.12, SD 0.3). In the HEM, a group characterized by high literacy but low empowerment is labeled as a needlessly dependent patient. However, the current profile’s eHealth literacy level, being below the average, cannot be considered high. Therefore, it is labeled as the “moderate-needs dependent patient.”

Profile 3 (68/2001, 3.4%) showed the lowest levels of eHealth literacy (mean 1.67, 95% CI 1.52-1.81, SD 0.62) and health empowerment (mean 2.33, 95% CI 2.14-2.51, SD 0.76), indicating substantial deficiencies in both literacy and empowerment. According to HEM, it is labeled as the “high-needs patient.”

Finally, profile 4 (529/2001, 26.4%) exhibited mean scores for eHealth literacy of 2.95 (95% CI 2.92-2.98, SD 0.41) and health empowerment of 3.95 (95% CI 3.92-3.97, SD 0.28). This suggests individuals in this profile possess a combination of low literacy with high empowerment, labeled as the “dangerous self-manager” according to the HEM.

Beyond having distinct combinations of eHealth literacy and empowerment levels, Welch ANOVA confirmed significant differences in the values of both eHealth literacy (*F*_3,287.48_=1103.55; *P*<.001) and health empowerment (*F*_3,289.77_=991.5; *P*<.001) among the 4 labeled profiles, with all pairwise comparisons reaching statistical significance (all *P*<.001, Table 2), confirming that these profiles are statistically distinct in both their combination patterns and magnitude levels of eHealth literacy and empowerment.

**Table 2:** Descriptive statistics and group comparisons for eHealth literacy and psychological health empowerment by patient profile^a^.

Variables	Profile 1 (n=996): Effective self-manager	Profile 2 (n=408): Moderate-needs dependent patient	Profile 3 (n=68): High-needs patient	Profile 4 (n=529): Dangerous self-manager	Welch ANOVA: *F* test (*df*)
eHealth literacy^b^, mean (SD)	3.98 (0.38)_A_	3.25 (0.41)_B_	1.67 (0.62)_C_	2.95 (0.41)_D_	1103.55 (3, 287.48)^c^
Health empowerment^b^, mean (SD)	4.05 (0.39)_A_	3.09 (0.30)_B_	2.33 (0.76)_C_	3.95 (0.28)_D_	991.50 (3, 289.77)^c^

^a^Welch ANOVA and Games-Howell post hoc tests were conducted due to the violation of the assumption of homogeneity of variance.

^b^Differing subscripts (A, B, C, D) indicate significant mean difference in a row at the significance level of *P*<.001.

^c^*P*<.001.

### Information Behavior Across Patient Profiles

Welch ANOVA indicated significant differences in intended information-seeking (*F*_3,289_=62.09; *P*<.001; η²=0.12) and intended disclosure (*F*_3,299.41_=66.08; *P*<.001; η²=0.09) across the 4 eHL-E Profiles (see Table 3).

Games-Howell post hoc tests provided detailed results for all pairwise comparisons. Regarding intended information-seeking, the “effective self-manager” group exhibited the highest intention (mean 4.01, 95% CI 3.96-4.06, SD 0.88), while the “high-needs patient” group had the lowest seeking intention (mean 2.13, 95% CI 1.83-2.44, SD 1.26). The “moderate-needs dependent patient” (mean 3.66, 95% CI 3.57-3.76, SD 0.98) and the “dangerous self-manager” (mean 3.69, 95% CI 3.61-3.78, SD 1.01) groups displayed similar seeking intentions. Except for the comparison between the “moderate-needs dependent patient” and the “dangerous self-manager” groups (mean difference –0.03, 95% CI –0.20 to 0.14; *P*=.97), all other mean differences were statistically significant at *P*<.001.

Regarding intended information disclosure, similar patterns emerged. The “effective self-manager” group once again had the greatest intention (mean 3.43, 95% CI 3.36-3.50, SD 1.02) to share online health information with their physicians. Conversely, the “high-needs patient” group exhibited the lowest intention to disclose (mean 1.85, 95% CI 1.6-2.1, SD 1). The “moderate-needs dependent patient” (mean 3, 95% CI 2.91-3.10, SD 0.98) and “dangerous self-manager” (mean 3.05, 95% CI 2.95-3.14, SD 1.12) groups were comparable. Except for the comparison between the “moderate-needs dependent patient” and the “dangerous self-manager” groups (mean difference 0.04, 95% CI –0.14 to 0.22; *P*=.93), all other group comparisons yielded significant mean differences (*P*<.001).

For actual information-seeking, the “effective self-manager” again had the highest proportion of individuals who reported seeking health information regarding their health condition before seeing the physician (800/996, 80.3%). The “moderate-needs dependent patient” group had a lower rate of 66.7% (272/408), and the “dangerous self-manager” group had a similar rate of 62.6% (331/529). In contrast, the “high-needs patient” group, characterized by the lowest eHealth literacy and empowerment, had the lowest rate, with only 36.8% (25/68) reporting actual seeking behavior. The chi-square test was significant (*χ*²_3_=103.4; *P*<.001) with a Cramer *V* of 0.227, indicating a moderate association (see Table 4). Pairwise comparisons with Bonferroni adjustment revealed that the “effective self-manager” had significantly higher seeking rates than all other profiles, while the “high-needs patient” had significantly lower rates than all other profiles. The “moderate-needs dependent patient” and “dangerous self-manager” profiles did not differ significantly from each other.

Among those who answered “yes” to actual seeking behavior, their actual disclosure behavior was examined (n=1428). The “effective self-manager” profile had a high proportion of individuals who disclosed health information (465/800, 58.1%). The “moderate-needs dependent patient” profile had a lower rate of 43.8% (119/272), and the “dangerous self-manager” profile had a similar rate of 47.4% (157/331). Interestingly, the “high-needs patient” profile showed the highest proportion of disclosures, with 64% (16/25) of those who searched for information online choosing to disclose it to their physicians. The chi-square test was significant (*χ*²_3_=23.1; *P*<.001) with a Cramer *V* of 0.127, suggesting a small to moderate association. Pairwise comparisons with Bonferroni adjustment showed that the “effective self-manager” profile had significantly higher disclosure rates than both the “moderate-needs dependent patient” and “dangerous self-manager” profiles (*P*<.05). These latter two profiles did not differ significantly from each other. The “high-needs patient” profile did not differ significantly from any other profile, which is likely due to the small sample size (n=16) limiting statistical power (Table 4).

**Table 3:** Descriptive statistics and group comparisons of intended information-seeking and disclosure behaviors across 4 patient profiles^a^.

Intended information behavior	Profile 1 (n=996): Effective self-manager, mean (SD)	Profile 2 (n=408): Moderate-needs dependent patient, mean (SD)	Profile 3 (n=68): High-needs patient, mean (SD)	Profile 4 (n=529): Dangerous self-manager, mean (SD)	Welch ANOVA: *F* test (*df*)
Intended seeking^b^	4.01 (0.88)_A_	3.66 (0.98)_B_	2.13 (1.26)_C_	3.69 (1.01)_B_	62.09 (3, 289)^c,d^
Intended disclosure^b^	3.43 (1.02)_A_	3.00 (0.98)_B_	1.85 (1.00)_C_	3.05 (1.12)_B_	66.08 (3, 299.41)^d,e^

^a^Welch ANOVA and Games-Howell post hoc tests were conducted due to the violation of the assumption of homogeneity of variance.

^b^Differing subscripts (A, B, C) indicate significant mean difference in a row at the significance level of *P*<.001.

^c^η^2^=0.12.

^d^*P*<.001.

^e^η^2^=0.09.

**Table 4:** Descriptive statistics and group comparisons of actual information-seeking and disclosure behaviors across 4 patient profiles^a^.

Actual information behaviors	Profile 1 (n=996): Effective self-manager, n (%)	Profile 2 (n=408): Moderate-needs dependent patient, n (%)	Profile 3 (n=68): High-needs patient, n (%)	Profile 4 (n=529): Dangerous self-manager, n (%)	Chi-square (*df*)
Actual seeking (yes)^b^	800 (80.3)_A_	272 (66.7)_B_	25 (36.8)_C_	331 (62.6)_B_	103.4 (3)^c,d^
Actual disclosure (yes)^b,e^	465 (58.1)_A_	119 (43.8)_B_	16 (64.0)_A,B_	157 (47.4)_B_	23.1 (3)^d,f^

^a^Different subscripts (A, B, C) within a row indicate significant differences between profiles at *P*<.05 or lower.

^b^Represents the frequency and percentage for dichotomous variable.

^c^Cramer *V*=0.227.

^d^*P*<.001.

^e^Only the participants who answered “yes” in actual seeking are included; hence, n=1428.

^f^Cramer *V*=0.127.

### The Role of PCC in Information Disclosure

We expect that controlling for patient predisposing (health empowerment) and enabling factors (eHealth literacy), PCC would be associated with patients’ disclosure of internet-sourced information to physicians. To examine this relationship, we conducted regression analyses. Intended disclosure showed no significant association with PCC (β=–0.007, 95% CI –0.09 to 0.07; *P*=.76). However, actual disclosure behavior demonstrated a positive association with PCC (odds ratio 1.26, 95% CI 1.04-1.53; *P*=.02). This indicates a positive relationship between PCC and patients’ disclosure of internet-sourced information to their physicians in medical encounters (see Table 5).

Two single moderator models using the Hayes PROCESS macro (model 1 with a multicategorical moderator) tested whether PCC related differently to information disclosure across the 4 patient-profile clusters. For intended disclosure (continuous outcome), the PCC × patient profile interaction was nonsignificant (*F*_3,1413_=0.24; *P*=.87); none of the 3 contrast-specific interaction terms was significant (|β|≤0.07, all *P*≥.5). The results were similar for actual disclosure (binary outcome): the interaction did not reach significance (*χ*²_3_=1.7; *P*=.64) and each individual interaction term was likewise nonsignificant (all *P*≥.33). These results indicate that the association between PCC and information disclosure did not significantly vary across patient profiles, suggesting a consistent relationship regardless of empowerment and literacy profiles.

**Table 5:** Multiple linear regression and logistic regression results for intended and actual information disclosure with physicians^a^.

Variables	Intended disclosure^b^			Actual disclosure^c^	
	Coefficients (95% CI)	*P* value	Odds ratio (95% CI)		*P* value
Age	0.00 (0.000 to 0.008)	.06	1.02^e^ (1.01 to 1.03)		.003
Gender	0.02 (–0.09 to 0.13)	.76	0.93 (0.72 to 1.21)		.59
Education	0.10^f^ (0.05 to 0.15)	<.001	1.01 (0.89 to 1.15)		.88
Health status	–0.04 (–0.11 to 0.03)	.27	1.19^g^ (1.01 to 1.39)		.04
Trust in online health information	0.18^f^ (0.10 to 0.26)	<.001	1.11 (0.92 to 1.34)		.30
eHealth literacy	0.31^f^ (0.22 to 0.40)	<.001	1.32^g^ (1.07 to 1.64)		.01
Health empowerment	0.20^f^ (0.10 to 0.30)	<.001	1.05 (0.82 to 1.34)		.69
PCC^d^	–0.01 (–0.09 to 0.07)	.76	1.26^g^ (1.04 to 1.53)		.02

^a^Mean, median, or mode imputation was used for missing values on age, gender, and education.

^b^*R*^2^=0.11.

^c^Nagelkerke *R*^2^=0.04.

^d^PCC: patient-centered communication.

^e^*P*<.01.

^f^*P*<.001.

^g^*P*<.05.

## Discussion

### Principal Results

This study identified 4 distinct patient profiles based on eHealth literacy and psychological health empowerment: effective self-managers, moderate-needs dependent patients, high-needs patients, and dangerous self-managers. These profiles demonstrated significantly different patterns in both online health information seeking prior to consultations and disclosure of this information to physicians. PCC was positively associated with actual information disclosure, and this association did not vary across patient profiles. Together, these findings demonstrate that eHealth literacy and empowerment create meaningful patient groups with distinct information behaviors, and PCC plays a consistent role in encouraging disclosure regardless of patient characteristics. Below, we discuss these findings in relation to existing literature and their implications for theory and practice.

First, our identification of 4 distinct patient profiles supports the HEM’s core premise that patients can be meaningfully categorized based on their literacy and empowerment levels. Three of these profiles—effective self-managers, high-needs patients, and dangerous self-managers—matched HEM predictions closely [24]. However, we found an interesting deviation. The HEM predicts a “needlessly dependent patient” with high literacy but low empowerment. Instead, we identified “moderate-needs dependent patients” who had both below-average literacy and low empowerment. This finding appears consistent with other empirical applications of HEM. For instance, a recent study classified 269 participants using depression literacy and empowerment; they only identified 2 of the 4 theoretical profiles [36]. Such findings suggest that while the HEM provides a valuable conceptual framework, researchers should anticipate that patient populations may not always exhibit all 4 theorized combinations. The specific profiles that emerge may depend on factors such as the targeted literacy used and the characteristics of the population of interest. For instance, when studying populations that are relatively educated and familiar with online technologies (thus having high eHealth literacy), the theoretically posited profiles of “high-needs patients” and “needlessly dependent patients” with low literacy may not be identified due to the overall high literacy baseline of the sample.

Interestingly, our sample split nearly evenly between “balanced” profiles (effective self-managers and high-needs patients, around 53%, 1064/2001 of the total sample) and “imbalanced” profiles where literacy and empowerment diverged (moderate-needs dependent patients and dangerous self-managers, around 47%, 937/2001). This finding suggests that eHealth literacy and psychological health empowerment do not necessarily develop in parallel. In practice, digital health initiatives aiming for patient empowerment often assume patients have the eHealth literacy needed to grasp their content [84], while online interventions that focus on improving patients’ literacy skills often treat empowerment as one of the primary outcomes [85]. Our findings suggest that the assumption of high eHealth literacy inherently leading to empowerment and effective patient engagement with eHealth resources may not hold true. Therefore, targeting health literacy alone may not guarantee corresponding improvements in empowerment and vice versa.

Second, we found significant behavioral differences across patient profiles. These patient profiles not just represent differences in their literacy and empowerment but also differ with their online health information seeking and disclosure patterns. One interesting finding emerged from the “high-needs patient” profile. They present a particularly intriguing information behavior pattern: while they showed the lowest information-seeking behavior, they demonstrated the highest disclosure rates when they did search. Rather than being passive or disengaged, “high-needs patients” appear highly selective in their information-seeking but deeply reliant on physician guidance. This suggests that when patients with low eHealth literacy and empowerment do seek information, they may place exceptional value on professional validation and interpretation.

The “dangerous self-manager” profile raises particular clinical concerns. These patients demonstrate moderately high information-seeking behavior but relatively low disclosure rates, creating a gap between information gathering and sharing with providers. Given their lower eHealth literacy levels, this combination of active seeking with limited disclosure could pose safety risks, as they may be making health decisions based on misinterpreted information without professional guidance.

In contrast, “effective self-managers” demonstrated the most comprehensive use of online health information, showing both high seeking behavior and high disclosure rates with physicians. This finding aligns with prior research linking high literacy and empowerment to more active health care participation and effective patient-provider collaboration [33,86].

Our analysis showed an interesting pattern regarding PCC and information disclosure. While participants’ retrospective assessments of PCC quality from past health care encounters showed no association with their intended disclosure behavior in hypothetical future scenarios, PCC was significantly linked to their actual disclosure behavior in those past encounters. This suggests that patients may form intentions about sharing online health information based on factors other than their previous communication experiences. However, the quality of physician communication during actual encounters does influence whether patients disclose their online findings in real time. Therefore, disclosure behavior appears more responsive to the immediate communication context rather than retrospective evaluations of past clinical experiences. This finding aligns with empirical studies showing that physicians’ communicative behavior during encounters affects whether patients share their online findings [21,66,87].

Furthermore, PCC demonstrated a statistically significant positive association with information disclosure behavior that was fairly consistent across all 4 patient profiles. This finding is particularly notable given the substantial information on behavioral differences we observed. This universal responsiveness suggests that PCC can promote engagement across diverse patient profiles, regardless of their health literacy or empowerment levels.

### Theoretical Implications

This study makes theoretical contributions to the understanding of patient eHealth literacy, empowerment, and the HEM. First, our findings provide empirical support for HEM. Our identification of patient profiles aligns closely with the model’s proposed combinations of high and low empowerment and health literacy. These profiles exhibited distinct information behaviors, from comprehensive information seeking and disclosure among “effective self-managers” to limited engagement among “high-needs patients” and “dangerous self-managers.” While previous HEM studies are primarily based on Western health contexts, our study demonstrates the model’s validity in Chinese health care settings. Additionally, our study extends HEM by integrating physician-patient communication as a contextual factor, which was not originally included in the model. Specifically, we demonstrated that PCC encourages patients to disclose online health information to their physicians, regardless of their empowerment or literacy combination. This finding indicates that future applications of HEM should incorporate interpersonal communication aspects as relevant contextual variables when examining patient health behaviors in clinical settings.

Second, our work advances the eHealth literacy and patient empowerment literature by demonstrating that both cognitive capabilities (eHealth literacy) and motivational agency (psychological health empowerment) are necessary for effective use of online health information. These constructs are often studied separately, yet our findings suggest they operate together. Future research should integrate both when modeling patient information behaviors to develop a more complete understanding of how patients access, evaluate, and act on health information.

### Practical Implications

Our findings offer practical insights into health education and clinical care. The identification of distinct eHL-E Profiles highlights the potential for tailored communication strategies for each patient group. Effective self-managers, with their high literacy and empowerment, can benefit from more autonomous decision-making opportunities and acknowledgment to continue engaging with reliable online health information. High-needs patients, in contrast, require interventions that enhance both their literacy skills and psychological empowerment; health care providers should therefore offer these patients greater support in navigating health information rather than solely expecting autonomous or shared decision-making. For dangerous self-managers, health programs should focus primarily on building literacy and information evaluation skills; providers should guide them in assessing online health information and encourage them to share and discuss their findings with physicians to prevent potential misuses.

In the digital age, patients commonly arrive at medical consultations with information gathered online [5]. Yet, our study, alongside works by others [88,89], highlights a concerning trend: patients often withhold this online-acquired information from their physicians. This reluctance to share online findings prevents opportunities to correct misconceptions from online searches and diminishes chances to better understand patients’ views and perceptions about their health. To encourage patients’ sharing, we recommend that physicians adopt PCC strategies. These include acknowledging patients’ efforts to search for information online as preparation for their visits rather than devaluing this information [90,91], asking open-ended questions about what patients have learned online, and encouraging patients to discuss any concerns about information they have found [15]. By fostering these open dialogues, patients can benefit from both the wealth of online health information and the expertise of medical professionals.

The study findings also have implications for digital health initiatives on eHealth literacy or empowerment. With the proliferation of digital tools and artificial intelligence (AI) technologies in health promotion [92,93], training programs should address both skill development and confidence-building. Specifically, digital health platforms can implement strategies such as differentiated information presentation based on users’ health literacy levels, gradually building eHealth literacy through scaffolded learning. Additionally, empowerment-focused features, such as personalized goal setting and progress tracking, can be integrated into online health applications to encourage patient participation.

### Limitations

Our study has several limitations. First, there is no established measure for estimating patient information disclosure behavior. Survey studies on this topic typically devised their own measures based on their specific focus [49]. The measurement of actual behavior, including information seeking and disclosure, relied on participants’ recall of previous medical visits with physicians. This could introduce recall bias to their responses.

Second, our sample was skewed toward young, highly educated individuals with college degrees or higher. This is also reflected in the overrepresentation of the effective self-manager group and underrepresentation of the high-needs patient group. The small size of the high-needs patient group may bias our results and limit their generalizability to those with low eHealth literacy and empowerment. The unbalanced sample may stem from our survey’s online distribution, which is more accessible to digital platform users. Future research should apply more inclusive sampling for those less digitally skilled. Additionally, our sample has a significant gender imbalance, with females representing approximately twice as many participants as males. This overrepresentation of female participants limits the generalizability of our findings across gender groups. Future studies should use stratified sampling methods to ensure more balanced gender representation.

Third, there is ongoing debate in the literature regarding health literacy measurement, which can be subjective (self-rated) or objective (performance-based) [94]. Our study used the eHEALS, which relies on self-reported perceptions of competence rather than objective assessment of actual skills. While eHEALS is widely validated and predictive of health behaviors and outcomes, we acknowledge that self-reported measures may not fully capture individuals’ actual eHealth literacy performance. Besides, there is growing recognition that traditional eHealth literacy measures like eHEALS may not fully address contemporary digital health challenges, and some advocate for a broader digital health literacy concept that encompasses the competence of using digital health tools and additional measurements to incorporate AI literacy. Future studies should consider including these newer measurements to capture the full spectrum of digital health competencies.

Fourth, our data were collected between September and November 2022, prior to the public release of ChatGPT (November 30, 2022) and before AI chatbots became widely available for health information seeking. Therefore, AI-generated health information use was not prevalent among participants and was not included in our study design. This represents a limitation, as the current digital health information landscape has evolved rapidly since our data collection. Future research should examine how AI literacy and the use of AI-generated health information shape patient-physician communication and information behavior patterns.

### Conclusions

This study extends existing literature by moving beyond online health information seeking to examine patients’ disclosure of online findings to physicians. Unlike prior research that examined eHealth literacy and psychological health empowerment separately, this study integrated these constructs to identify meaningful patient profiles with distinct information behavior patterns. Findings indicate that active health information seeking and disclosure require both adequate eHealth literacy skills and psychological health empowerment. Furthermore, PCC facilitates disclosure regardless of patient profile. For practice, physicians should adopt a PCC that acknowledges patients’ online research efforts and actively supports their eHealth literacy and empowerment. This can create a clinical environment where patients feel comfortable sharing their online findings, ultimately contributing to safer use of online health information and stronger patient-physician relationships.

## Appendix

Multimedia Appendix 1 Complete measurement scales.

## Abbreviations

AI: artificial intelligence
CHERRIES: Checklist for Reporting Results of Internet e-Surveys
eHEALS: eHealth Literacy Scale
eHL-E Profiles: eHealth Literacy and Empowerment Profiles
HEM: Health Empowerment Model
HINTS-China: Health Information National Trends Survey in China
PCC: patient-centered communication
PHES: Psychological Health Empowerment Scale
RQ: research question

## Data Availability

The dataset used in this study is available from the corresponding author on reasonable request.
