# Supplementary material for: Seeking But Not Discussing Online Health Information With Physicians: Cross-Sectional Survey Study of eHealth Literacy–Empowerment Profiles and Patient-Centered Communication
Source: J Med Internet Res. 2026 Feb 18;28:e78836. doi: 10.2196/78836 (PMC12961392; doi:10.2196/78836)
Supplement: Multimedia Appendix 1 [file jmir_v28i1e78836_app1.docx]

**Multimedia Appendix 1: Complete Measurement Scales**

**1. eHealth Literacy Scale (eHEALS)**
Items: 8
Response Scale: 5-point Likert (1 = Strongly disagree to 5 = Strongly agree)
Scoring: Mean of all items (higher = greater e-health literacy)

| **Item** | **Survey Wording** |
| --- | --- |
| 1 | I know how to find helpful health resources on the Internet. |
| 2 | I know how to use the Internet to answer my health questions. |
| 3 | I know what health resources are available on the Internet. |
| 4 | I know where to find helpful health resources on the Internet. |
| 5 | I know how to use the health information I find on the Internet to help me. |
| 6 | I have the skills to evaluate the health resources I find on the Internet. |
| 7 | I can tell high quality from low-quality health resources on the Internet. |
| 8 | I feel confident in using information from the Internet to make health decisions. |

**2. Psychological Health Empowerment Scale (PHES)**
Items: 8
Response Scale: 5-point Likert (1 = Strongly disagree to 5 = Strongly agree)
Scoring: Mean of all items (higher = greater psychological health empowerment)

| Item | **Survey Wording** |
| --- | --- |
| 1 | I will use the necessary means and goods to manage health effectively. |
| 2 | I can understand my disease better than anyone. |
| 3 | I can motivate myself to manage my health and make a better life. |
| 4 | I can make every possible effort to achieve health goals. |
| 5 | I am enthusiastic about my efforts to manage my health. |
| 6 | I know where I can ask for help to manage my disease. |
| 7 | I can manage my disease conditions. |
| 8 | I can make a realistic health plan. |

**3. Patient-Centered Communication Scale**

Items: 7
Response Scale: 5-point Likert (1 = Never to 5 = Always)
Scoring: Mean of all items (higher = better patient-centered communication)

Question Stem: "The following questions concern your communication with all doctors, nurses, or other health professionals you saw during the past 12 months. How often did they do each of the following:"

| **Item** | **Survey Wording** |
| --- | --- |
| 1 | Give me the chance to ask all the health-related questions I had. |
| 2 | Give the attention I needed to my feelings and emotions. |
| 3 | Involve me in decisions about my health care as much as I want. |
| 4 | Ensure I understood what I needed to do to take care of my health. |
| 5 | Explain things in a way I could understand. |
| 6 | Spend enough time with me. |
| 7 | Help me deal with feelings of uncertainty about my health or health care. |

**4. Health Information Seeking and Disclosure Behavior**

**4A. Actual Seeking Behavior**

Items: 1
Response: Binary (Yes = 1, No or "I am not sure" = 0)

| **Item** | **Wording** |
| --- | --- |
| Actual Seeking | Reflecting on your recent medical consultations, did you search for health information on the Internet about your condition before meeting with your doctor? |

**4B. Actual Disclosure Behavior**

Items: 1
Response Format: Binary (Yes = 1, No or "I am not sure" = 0)

| **Item** | **Wording** |
| --- | --- |
| Actual Disclosure | Furthermore, did you discuss the information you found on the Internet with your doctor during the consultation? |

**4C. Intended Seeking Behavior**

Items: 1
Response Scale: 5-point Likert (1 = Very unlikely to 5 = Very likely)

Scenario: "Imagine you had chest pain for one week and never had this symptom before. Therefore, you decide to make a medical appointment with a doctor you never met before."

| **Item** | **Wording** |
| --- | --- |
| Intended Seeking | How likely will you search health information for your condition on the Internet before visiting the doctor? |

**4D. Intended Disclosure Behavior**

Items: 1
Response Scale: 5-point Likert (1 = Very unlikely to 5 = Very likely)

Scenario: (Same as 4C)

| **Item** | **Wording** |
| --- | --- |
| Intended Disclosure | How likely will you share the information you found on the Internet with the doctor? |

**5. Control Variables**

**5A. Self-Rated General Health Status**
Items: 1
Response Scale: 5-point scale (1 = Poor, 2 = Fair, 3 = Good, 4 = Very good, 5 = Excellent)

| **Item** | **Wording** |
| --- | --- |
| Health Status | In general, would you say your health is: |

**5B. Trust in Online Health Information**
Items: 3 (from online sources)
Response Scale: 5-point scale (1 = Not at all to 5 = A lot)
Scoring: Mean of all items (higher = greater trust)

Question Stem: "In general, how much would you trust health or medical information from each of the following?"

| **Item** | **Information Source** |
| --- | --- |
| 1 | Websites or Search engines |
| 2 | Applications: Health-related or News applications |
| 3 | Social media: WeChat/Weibo/QQ/Blogs or forums |

**5C. Demographic Variables**

| **Variable** | **Measurement** |
| --- | --- |
| Age | Continuous in years |
| Gender | Binary: Female = 0, Male = 1 |
| Education | 6-point ordinal scale: 1 = Primary school and below, 2 = Junior middle school, 3 = High school, 4 = Junior college, 5 = Bachelor's degree, 6 = Higher than Bachelor's degree |

**Note:** All items were administered in Chinese. English wordings presented here are back-translations for reference.
